# *N*-Ethyl-6-ethyl­amino-4-oxo-1,3,5-triazin-2-aminium chloride (Oxysimazine·HCl)

**DOI:** 10.1107/S1600536810033775

**Published:** 2010-09-04

**Authors:** J. Emery Brown, Russell G. Baughman

**Affiliations:** a Department of Chemistry, Truman State University, Kirksville, MO 63501-4221, USA

## Abstract

In the title molecular salt, C_7_H_14_N_5_O^+^·Cl^−^ (the HCl salt of the oxo derivative of the triazine herbicide simazine), the cation and anion are linked by N—H⋯Cl hydrogen bonds. The chloride ion is also involved in a close electrostatic inter­action with an inversion-related triazine ring [Cl⋯centroid distance = 3.201 (1) Å]. A π–π inter­action having a centroid⋯centroid distance of 3.456 (2) Å exists between pairs of rings *via* another inversion relation. The triazine ring and adjacent non-H atoms are essentially planar (r.m.s. deviation = 0.042 Å), while both methyl groups are approximately perpendicular and on the same side of the plane [torsion angles = 79.3 (3) and −84.6 (3)°]. Upon exposure to X-rays for about two days, the color of the title compound changed from colorless to a pale yellow-orange with no apparent affect on the structure as evidenced by no significant change in the intensities of the standard reflections.

## Related literature

The structure determinations of twoherbicides have been reported (Black & Baughman, 2010 ▶; Baughman & Yu, 1988 ▶ and references cited therein) as has information on the mode of action of this class of herbicides (Roberts, 1998 ▶). For the Gaussian calculation, see: Frisch *et al.* (2009 ▶).
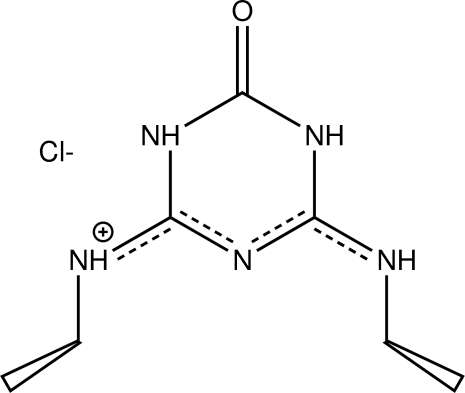


## Experimental

### 

#### Crystal data

C_7_H_14_N_5_O^+^·Cl^−^
*M* *_r_* = 219.68
Orthorhombic, 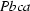

*a* = 8.2804 (5) Å
*b* = 14.5930 (9) Å
*c* = 17.9924 (11) Å
*V* = 2174.1 (2) Å^3^
*Z* = 8
Mo *K*α radiation
μ = 0.33 mm^−1^
*T* = 295 K
0.40 × 0.28 × 0.27 mm

#### Data collection

Bruker P4 diffractometer
Absorption correction: integration (*XSHELL*; Bruker, 1999 ▶) *T*_min_ = 0.912, *T*_max_ = 0.927
2548 measured reflections
1969 independent reflections
1316 reflections with *I* > 2σ(*I*)
*R*_int_ = 0.026
3 standard reflections every 100 reflections  intensity decay: 1.1%

#### Refinement

*R*[*F* ^2^ > 2σ(*F* ^2^)] = 0.037
*wR*(*F* ^2^) = 0.094
*S* = 1.02
1940 reflections
130 parameters
H-atom parameters constrained
Δρ_max_ = 0.16 e Å^−3^
Δρ_min_ = −0.15 e Å^−3^

### 

Data collection: *XSCANS* (Bruker, 1996 ▶); cell refinement: *XSCANS*; data reduction: *XSCANS*; program(s) used to solve structure: *SHELXS86* (Sheldrick, 2008 ▶); program(s) used to refine structure: *SHELXL97* (Sheldrick, 2008 ▶); molecular graphics: *SHELXTL/PC* (Sheldrick, 2008 ▶); software used to prepare material for publication: *SHELXTL/PC* and *SHELXL97*.

## Supplementary Material

Crystal structure: contains datablocks I, global. DOI: 10.1107/S1600536810033775/fl2312sup1.cif

Structure factors: contains datablocks I. DOI: 10.1107/S1600536810033775/fl2312Isup2.hkl

Additional supplementary materials:  crystallographic information; 3D view; checkCIF report

## Figures and Tables

**Table 1:** Hydrogen-bond geometry (Å, °)

*D*—H⋯*A*	*D*—H	H⋯*A*	*D*⋯*A*	*D*—H⋯*A*
N1—H1*A*⋯Cl1	0.86	2.43	3.225 (2)	154
N2—H2*A*⋯Cl1^i^	0.86	2.24	3.080 (2)	167
N4—H4*C*⋯Cl1	0.86	2.44	3.246 (2)	156
N5—H5*D*⋯Cl1^i^	0.86	2.80	3.536 (2)	144
C5—H5*A*⋯O1^ii^	0.96	2.53	3.471 (4)	166

Symmetry codes: (i) 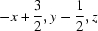; (ii) 
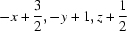
.

**Fig. 1.**
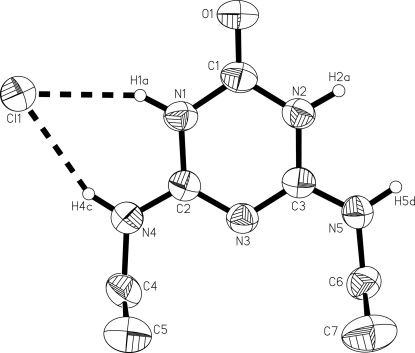
The asymmetric unit of the title compound, showing the labeling of the non-H atoms and H atoms involved in intramolecular hydrogen bonding (dashed lines). Displacement ellipsoids are drawn at the 50% probability levels and H atoms are drawn as small spheres of arbitrary radius.

**Fig. 2.**
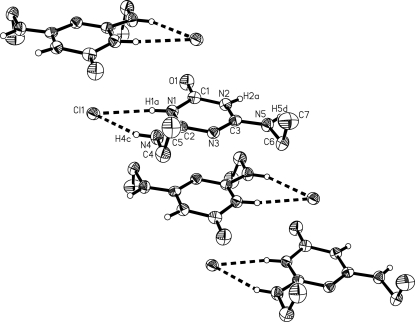
View of inversion-related molecules at (1-x, 1-y, 1-z) and (2-x, 1-x, 1-z) showing Cl···ring and ring···ring (π–π) interactions. Only 4 Cl's are shown for charge balance; four others (not shown) are H-bonded to H2 and H5-type H's on the periphery of this view. To minimize congestion, displacement ellipsoids are drawn at the 30% probability levels.

## supplementary crystallographic information

### Comment

During an attempt to crystallize simazine (C_7_H_12_ClN_5_) from a dilute HCl_(aq)_ solution exposed to room light, the title compound was produced. The crystal structure of the HCl salt of the oxo derivative of simazine was determined as part of a larger project involving the structure determinations of herbicides (Black & Baughman, 2010; Baughman and Yu, 1988 and references cited therein). Simazine is a selective systemic herbicide that inhibits photosynthesis in annual grasses and broad-leaved weeds (Roberts, 1998).

Two intramolecular hydrogen bonds involve the chloride ion (Cl1) with H1a and H4c (Table 1 and Figs. 1 & 2). The same chloride ion is also intermolecularly bonded to the H2a and H5d H atoms present in a symmetry-related molecule. A weak intermolecular interaction was observed between H5a and O1 in another symmetry-related molecule. See Table 1 for the various symmetry operations.

The cation displays a pseudo-vertical mirror perpendicular to Fig. 1 and going through O1, C1, and N3. The C2—N1, N1—C1, C1—N2, and N2—C3 bond lengths are all within 2σ of each other, yet, on the average, are 15σ longer than the C2—N3 and N3—C3 bond lengths which are within 2σ of each other. The values of the torsion angles C2—N4—C4—C5 [79.3 (3)°] and C3—N5—C6—C7 [-84.6 (3)°] show that the ethyl groups are pointed by a comparable amount in the same direction above the triazine ring.

Results from a Gaussian (Frisch *et al.*, 2009) single point calculation at the M06–2X/6–31 G(*d*) level of theory indicate that the bond orders are largely consistent with the localized structure shown in the Scheme. All three C's in the ring have partially positive charges, with the one on C1 being the largest (+0.85 *vs*. +0.70 e^-^ for C2 & C3). Since the crystals are initially colorless, then turn to a pale yellow-orange after ~2 days exposure to X-rays, the close [3.201 (1) Å] interactions (Fig. 2) of Cl1···Centroid of the ring at 2 - *x*, 1 - *y*, 1 - *z*) are likely just electrostatic in nature rather than charge-transfer based. However, an interplanar distance of 3.24 (3) Å indicates that a π–π interaction (Fig. 2) exists between pairs of rings *via* 1 - *x*, 1 - *y* 1 - *z*. This may be the source of the observed color change.

The conversion of simazine to its oxo derivative occurred during the slow evaporation of an HCl_(aq)_ solution in the presence of light. FT—IR analyses of solid samples of the original simazine and the title compound show a C—Cl stretch only for simazine and a C═O stretch only for the salt. The light may have initiated a free-radical reaction between the chlorine in the original simazine and H_2_O which produced the derivative. In field applications, sunlight might then convert the simazine to the oxo derivative in the presence of acidic soil. The bioactivity of simazine, or other triazines (*e.g.*, the widely-used atrazine), may involve a different free-radical reaction or the oxo versions of the compounds are the active agents.

### Experimental

Simazine (99.9%), the parent compound, was purchased from Sigma-Aldrich (Riedel-de Haën) and used without further purification. Crystals of the title compound were grown by slow evaporation of a solution in dilute HCl_(aq)_.

### Refinement

Approximate positions of the all of the H's were first obtained from a difference map, then placed into "ideal" positions and refined as a rotational group. Bond lengths were constrained at 0.93 Å (AFIX 43) for aromatic C—H's; at 0.96 Å (AFIX 137) for methyl C—H's; at 0.97 Å (AFIX 137) for ethyl C—H's; and at 0.86 Å (AFIX 43) for N—H's. *U*_iso_(H) were fixed at 1.5*U*_eq_(parent) for OH and methyl H's, and 1.2 *U*_eq_(parent) for all other H's.

In the final stages of refinement 11 very small or negative *F*_o_'s were deemed to be in high disagreement with their *F*_c_'s and were eliminated from final refinement.

For the set of standard reflection *I*'s monitored during data collection, an average and standard deviation were calculated, as was the ratio of this standard deviation to the average *I* for each standard. The average of these 3 ratios, expressed as a percentage, is reported.

### Crystal data

**Table:**

C_7_H_14_N_5_O^+^·Cl^−^	*F*(000) = 928
*M**_r_* = 219.68	*D*_x_ = 1.342 Mg m^−^^3^
Orthorhombic, *P**b**c**a*	Mo *K*α radiation, λ = 0.71073 Å
Hall symbol: -P 2ac 2ab	Cell parameters from 100 reflections
*a* = 8.2804 (5) Å	θ = 10.9–20.4°
*b* = 14.5930 (9) Å	µ = 0.33 mm^−^^1^
*c* = 17.9924 (11) Å	*T* = 295 K
*V* = 2174.1 (2) Å^3^	Parallelpiped, colorless
*Z* = 8	0.40 × 0.28 × 0.27 mm

### Data collection

**Table:**

Bruker P4 diffractometer	1316 reflections with *I* > 2σ(*I*)
Radiation source: normal-focus sealed tube	*R*_int_ = 0.026
graphite	θ_max_ = 25.3°, θ_min_ = 2.3°
θ/2θ scans	*h* = −9→1
Absorption correction: integration (XSHELL; Bruker, 1999)	*k* = −1→17
*T*_min_ = 0.912, *T*_max_ = 0.927	*l* = −1→21
2548 measured reflections	3 standard reflections every 100 reflections
1969 independent reflections	intensity decay: A value of 1.1% for the average ratio of σ(*I*):(average *I*) for the three standards.

### Refinement

**Table:**

Refinement on *F*^2^	Secondary atom site location: difference Fourier map
Least-squares matrix: full	Hydrogen site location: inferred from neighbouring sites
*R*[*F*^2^ > 2σ(*F*^2^)] = 0.037	H-atom parameters constrained
*wR*(*F*^2^) = 0.094	*w* = 1/[σ^2^(*F*_o_^2^) + (0.0341*P*)^2^ + 0.8482*P*] where *P* = (*F*_o_^2^ + 2*F*_c_^2^)/3
*S* = 1.02	(Δ/σ)_max_ = 0.001
1940 reflections	Δρ_max_ = 0.16 e Å^−^^3^
130 parameters	Δρ_min_ = −0.15 e Å^−^^3^
0 restraints	Extinction correction: *SHELXL97* (Sheldrick, 2008), *F*_c_*=k*F*_c_[1+0.001x*F*_c_^2^λ^3^/sin(2θ)]^-1/4^
Primary atom site location: structure-invariant direct methods	Extinction coefficient: 0.0061 (6)

### Special details

**Table:**

Geometry. All s.u.'s (except the s.u. in the dihedral angle between two l.s. planes) are estimated using the full covariance matrix. The cell s.u.'s are taken into account individually in the estimation of s.u.'s in distances, angles and torsion angles; correlations between s.u.'s in cell parameters are only used when they are defined by crystal symmetry. An approximate (isotropic) treatment of cell s.u.'s is used for estimating s.u.'s involving l.s. planes.
Refinement. Refinement of *F^2^* against ALL reflections. The weighted *R*-factor, w*R*, and goodness of fit, *S*, are based on *F^2^*, conventional *R*-factors, *R*, are based on *F*, with *F* set to zero for negative *F^2^*. The threshold expression of *F^2^* > 2σ(*F^2^*) is used only for calculating *R*-factors(gt) *etc*. and is not relevant to the choice of reflections for refinement. *R*-factors based on *F^2^* are statistically about twice as large as those based on *F*, and *R*-factors based on ALL data will be even larger.

### Fractional atomic coordinates and isotropic or equivalent isotropic displacement parameters (Å^2^)

**Table:**

	*x*	*y*	*z*	*U*_iso_*/*U*_eq_	
Cl1	0.99717 (7)	0.66337 (4)	0.44093 (3)	0.0479 (2)	
O1	0.7993 (2)	0.37960 (13)	0.38752 (9)	0.0624 (5)	
N1	0.7703 (2)	0.48903 (13)	0.47604 (9)	0.0416 (5)	
H1A	0.8280	0.5280	0.4519	0.050*	
N2	0.6260 (2)	0.35562 (13)	0.48312 (10)	0.0413 (5)	
H2A	0.5908	0.3055	0.4639	0.050*	
N3	0.6097 (2)	0.46066 (12)	0.58214 (10)	0.0402 (5)	
N4	0.7645 (3)	0.59006 (13)	0.57336 (10)	0.0499 (5)	
H4C	0.8258	0.6245	0.5467	0.060*	
N5	0.4670 (2)	0.32663 (13)	0.58517 (11)	0.0461 (5)	
H5D	0.4422	0.2757	0.5640	0.055*	
C1	0.7374 (3)	0.40559 (16)	0.44432 (13)	0.0443 (6)	
C2	0.7138 (3)	0.51210 (15)	0.54523 (12)	0.0387 (5)	
C3	0.5681 (3)	0.38174 (16)	0.55095 (12)	0.0384 (5)	
C4	0.7206 (4)	0.62068 (19)	0.64822 (14)	0.0600 (7)	
H4A	0.6073	0.6077	0.6570	0.072*	
H4B	0.7359	0.6864	0.6518	0.072*	
C5	0.8195 (4)	0.5745 (2)	0.70630 (15)	0.0788 (10)	
H5A	0.7892	0.5972	0.7544	0.118*	
H5B	0.9318	0.5871	0.6977	0.118*	
H5C	0.8014	0.5096	0.7042	0.118*	
C6	0.3943 (3)	0.34737 (18)	0.65733 (12)	0.0505 (6)	
H6A	0.2925	0.3149	0.6615	0.061*	
H6B	0.3714	0.4125	0.6599	0.061*	
C7	0.4998 (4)	0.3215 (2)	0.72113 (15)	0.0802 (10)	
H7A	0.4479	0.3384	0.7669	0.120*	
H7B	0.6012	0.3530	0.7171	0.120*	
H7C	0.5181	0.2566	0.7205	0.120*	

### Atomic displacement parameters (Å^2^)

**Table:**

	*U*^11^	*U*^22^	*U*^33^	*U*^12^	*U*^13^	*U*^23^
Cl1	0.0491 (3)	0.0455 (3)	0.0491 (3)	0.0041 (3)	0.0000 (3)	0.0024 (3)
O1	0.0758 (13)	0.0704 (12)	0.0409 (9)	−0.0070 (11)	0.0160 (10)	−0.0152 (9)
N1	0.0469 (12)	0.0426 (11)	0.0352 (10)	0.0009 (9)	0.0028 (9)	0.0033 (9)
N2	0.0429 (11)	0.0423 (11)	0.0387 (10)	−0.0019 (10)	−0.0029 (9)	−0.0069 (9)
N3	0.0397 (11)	0.0419 (10)	0.0390 (9)	0.0006 (9)	0.0019 (9)	−0.0032 (9)
N4	0.0600 (13)	0.0450 (12)	0.0449 (11)	−0.0072 (11)	0.0063 (11)	−0.0032 (10)
N5	0.0458 (13)	0.0460 (11)	0.0466 (10)	−0.0068 (10)	0.0017 (9)	−0.0033 (9)
C1	0.0483 (14)	0.0479 (14)	0.0368 (12)	0.0032 (12)	−0.0040 (12)	−0.0026 (12)
C2	0.0392 (13)	0.0393 (13)	0.0376 (12)	0.0035 (11)	−0.0030 (11)	0.0001 (10)
C3	0.0352 (12)	0.0447 (13)	0.0354 (11)	0.0051 (11)	−0.0048 (10)	−0.0002 (11)
C4	0.0691 (19)	0.0547 (15)	0.0563 (15)	−0.0076 (15)	0.0082 (15)	−0.0177 (14)
C5	0.097 (2)	0.091 (2)	0.0476 (16)	−0.010 (2)	−0.0022 (17)	−0.0057 (16)
C6	0.0467 (14)	0.0576 (15)	0.0473 (13)	−0.0061 (13)	0.0062 (12)	0.0008 (12)
C7	0.091 (2)	0.101 (3)	0.0486 (15)	0.023 (2)	−0.0016 (17)	0.0070 (16)

### Geometric parameters (Å, °)

**Table:**

O1—C1	1.204 (3)	N5—H5D	0.8600
N1—C2	1.372 (3)	C4—C5	1.489 (4)
N1—C1	1.372 (3)	C4—H4A	0.9700
N1—H1A	0.8600	C4—H4B	0.9700
N2—C3	1.366 (3)	C5—H5A	0.9600
N2—C1	1.368 (3)	C5—H5B	0.9600
N2—H2A	0.8600	C5—H5C	0.9600
N3—C2	1.322 (3)	C6—C7	1.491 (4)
N3—C3	1.327 (3)	C6—H6A	0.9700
N4—C2	1.314 (3)	C6—H6B	0.9700
N4—C4	1.465 (3)	C7—H7A	0.9600
N4—H4C	0.8600	C7—H7B	0.9600
N5—C3	1.314 (3)	C7—H7C	0.9600
N5—C6	1.463 (3)		
			
C2—N1—C1	121.8 (2)	N4—C4—H4A	109.3
C2—N1—H1A	119.1	C5—C4—H4A	109.3
C1—N1—H1A	119.1	N4—C4—H4B	109.3
C3—N2—C1	123.0 (2)	C5—C4—H4B	109.3
C3—N2—H2A	118.5	H4A—C4—H4B	107.9
C1—N2—H2A	118.5	C4—C5—H5A	109.5
C2—N3—C3	116.73 (19)	C4—C5—H5B	109.5
C2—N4—C4	122.6 (2)	H5A—C5—H5B	109.5
C2—N4—H4C	118.7	C4—C5—H5C	109.5
C4—N4—H4C	118.7	H5A—C5—H5C	109.5
C3—N5—C6	123.5 (2)	H5B—C5—H5C	109.5
C3—N5—H5D	118.3	N5—C6—C7	113.0 (2)
C6—N5—H5D	118.3	N5—C6—H6A	109.0
O1—C1—N2	123.5 (2)	C7—C6—H6A	109.0
O1—C1—N1	123.2 (2)	N5—C6—H6B	109.0
N2—C1—N1	113.3 (2)	C7—C6—H6B	109.0
N4—C2—N3	120.4 (2)	H6A—C6—H6B	107.8
N4—C2—N1	116.9 (2)	C6—C7—H7A	109.5
N3—C2—N1	122.6 (2)	C6—C7—H7B	109.5
N5—C3—N3	119.9 (2)	H7A—C7—H7B	109.5
N5—C3—N2	118.1 (2)	C6—C7—H7C	109.5
N3—C3—N2	122.0 (2)	H7A—C7—H7C	109.5
N4—C4—C5	111.8 (2)	H7B—C7—H7C	109.5
			
C3—N2—C1—O1	173.6 (2)	C1—N1—C2—N3	−8.0 (3)
C3—N2—C1—N1	−7.2 (3)	C6—N5—C3—N3	0.4 (3)
C2—N1—C1—O1	−171.7 (2)	C6—N5—C3—N2	−178.9 (2)
C2—N1—C1—N2	9.1 (3)	C2—N3—C3—N5	179.0 (2)
C4—N4—C2—N3	5.1 (4)	C2—N3—C3—N2	−1.8 (3)
C4—N4—C2—N1	−176.6 (2)	C1—N2—C3—N5	−176.8 (2)
C3—N3—C2—N4	−178.1 (2)	C1—N2—C3—N3	3.9 (3)
C3—N3—C2—N1	3.8 (3)	C2—N4—C4—C5	79.3 (3)
C1—N1—C2—N4	173.9 (2)	C3—N5—C6—C7	−84.6 (3)

### Hydrogen-bond geometry (Å, °)

**Table:**

*D*—H···*A*	*D*—H	H···*A*	*D*···*A*	*D*—H···*A*
N1—H1A···Cl1	0.86	2.43	3.225 (2)	154
N2—H2A···Cl1^i^	0.86	2.24	3.080 (2)	167
N4—H4C···Cl1	0.86	2.44	3.246 (2)	156
N5—H5D···Cl1^i^	0.86	2.80	3.536 (2)	144
C5—H5A···O1^ii^	0.96	2.53	3.471 (4)	166

Symmetry codes: (i) −*x*+3/2, *y*−1/2, *z*; (ii) −*x*+3/2, −*y*+1, *z*+1/2.
